# Step by Step about Germ Cells Development in Canine

**DOI:** 10.3390/ani11030598

**Published:** 2021-02-25

**Authors:** Aline Fernanda de Souza, Naira Caroline Godoy Pieri, Daniele dos Santos Martins

**Affiliations:** Department of Veterinary Medicine, Faculty of Animal Sciences and Food Engineering, University of São Paulo, Pirassununga 13635-900, Brazil; alinsouza25@gmail.com (A.F.d.S.); nairagodoy@gmail.com (N.C.G.P.)

**Keywords:** canine, primordial germ cells, spermatogonial stem cells

## Abstract

**Simple Summary:**

The progression of germ cells is a remarkable event that allows biological discovery in the differ-entiation process during in vivo and in vitro development. This is crucial for understanding one toward making oogenesis and spermatogenesis. Companion animals, such as canine, could offer new animal models for experimental and clinical testing for translation to human models. In this review, we describe the latest and more relevant findings on germ cell development. In addition, we showed the methods available for obtaining germ cells in vitro and the characterization of pri-mordial germ cells and spermatogonial stem cells. However, it is necessary to further conduct basic research in canine to clarify the beginning of germ cell development.

**Abstract:**

Primordial germ cells (PGCs) have been described as precursors of gametes and provide a connection within generations, passing on the genome to the next generation. Failures in the formation of gametes/germ cells can compromise the maintenance and conservation of species. Most of the studies with PGCs have been carried out in mice, but this species is not always the best study model when transposing this knowledge to humans. Domestic animals, such as canines (canine), have become a valuable translational research model for stem cells and therapy. Furthermore, the study of canine germ cells opens new avenues for veterinary reproduction. In this review, the objective is to provide a comprehensive overview of the current knowledge on canine germ cells. The aspects of canine development and germ cells have been discussed since the origin, specifications, and development of spermatogonial canine were first discussed. Additionally, we discussed and explored some in vitro aspects of canine reproduction with germ cells, such as embryonic germ cells and spermatogonial stem cells.

## 1. Introduction

The renewal capacity of germ cells to originate from descending cell lines (gametes) has been described for more than a decade, but the effectiveness of characterizing and capitalizing on its resources and potential is still recent [1]. However, the effectiveness of characterizing and capitalizing on its resources and potentials is still recent. During the development of the germ cells, the genome’s regulation, such as specific genetic and epigenetic factors, is essential to guide the specification and enhance the gametogenic lineage [2]. The transcription mechanism of the primordial germ cell until the differentiation of male and female gametes is different for each species. Several germ cell studies are directed to mice species due to the animal being easily manipulated. However, research with germ cells from other animal models within the niche of domestic animals, such as canids, could expand knowledge. The canine model has physiology and genetics similar to the human species [3]. Furthermore, the collection of materials for the study of germ cells can be obtained through canine neutering or spaying in veterinary hospitals.

Additionally, previous studies in humans, mice, and domestic animals showed that it is possible to immortalize the germ cells through in vitro cultivation [4]. This type of differentiation becomes essential, as it expands the possibilities of using germ cells in cell therapies and differentiating gametes in vitro. The production of gametes in vitro can help to understand the mechanisms that can cause infertility due to the loss of germ cells at some point in their development. In the future, it may help in the preservation of endangered species. Even though germ cells’ study could bring a new advance in animal reproduction, domestic animals’ research is not frequently as in mice and humans. Improvement of in vitro germ cell production in domestic animals would help understand each animal’s specific reproductive biology.

In canines, most studies have focused on in vitro spermatogonial stem cells (SSCs) showing the ability to restore and produce germ cells [5]. The SSCs in vitro were able to originate other cell lines as spermatocytes and spermatids until they became sperm. This pioneering study opens a field of research with a focus on reproductive biology based on development mechanisms and the functions of germ stem cell investigations in the restoration of fertility [6]. In addition, research has also shown the production of embryonic germ cells (EGCs), and primordial germ cells like in canines [7,8]. The canine model could open up opportunities to discover new signaling molecules, transcription factors, mechanisms of the self-renew cell, and differentiation that are important for the development of germ cells.

Research related to the development and in vitro production of germ cells in domestic animals would help to understand the specific reproductive biology of each animal. In summary, this review explored the most relevant research on canine germ cells’ development in vivo. In addition, we discuss the progress in germ cell induction in vitro and its applicability in reproductive veterinary medicine.

## 2. Embryological and Primordial Germ Cells Development

Domestic canines descend from the wild wolf which was domesticated by humans, originating the many canine breeds found currently. Thus, canines have become essential to humans as pets, for protection, and aid in hunting. In medicine, canines have been used as an animal model of disorders or diseases that affect humans because canines share genetic disorders and chronic diseases with humans [9]. The beginning of the timeline of fertilization and embryonic development of a canine is similar to that of other mammals. The canine conceptus measures around 1.2–1.5 mm 16 days post-fertilization, the development event is characterized by the ectodermal formation of the neural tube, and first somites segments [10]. At 17 dpf, the embryo showed the formation of three primary cerebral vesicles, eight pairs of somites, and an optic vesicle. Placentation and embryonic annexes (allantois and amnion) developed around 17–18 dpf [11]. In addition, it was observed that around 21–22 dpf, the amnion was fully formed with other annexes of the placenta [11]. During 22 dpf, the external appearance of the embryo is changed head and tail come close together, so the embryo forms a “C” shape. There is a growth up of the limbs and it is possible to detect the third pharyngeal arch. The respiratory, digestive, and urinary system is in development, being possible to observe the primitive kidney: mesonephros [12,13]. In addition, around 20–21 dpf primordial germ cells (PGCs) migrate through the mesenchyme between the celomic epithelium to gonadal ridge [14].

**Figure 1:**
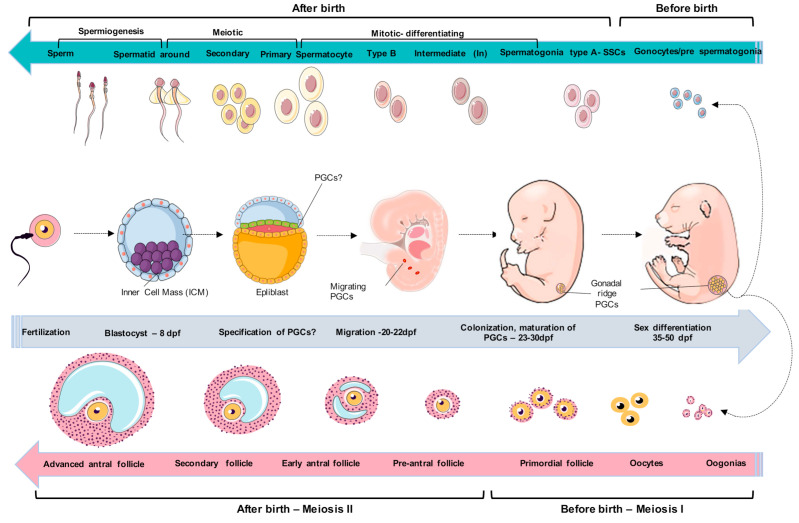
Schematic model of canine germ-cell development. After implantation of blastocysts, some canine germ cells will emerge; however, they could be around the amnion or the epiblast. Canine primordial germ cells (PGCs) undergo migration for 20–22 days post-fertilization (dpf) through developing hindgut and mesentery and colonize the genital ridges during 23–25 dpf. The canine PGCs enter the cell maturation process around 27–30 dpf. The period around 35–40 dpf is marked by the initiation of sexual differentiation, during which the cells can be morphologically distinguished as males or females. The male gonads undergo major morphological changes, medullary cords are differentiated into seminiferous cords. The female gonadal ridge was divided into the medulla (inside the gonad) and cortex (outside of the gonad). Around 45–55 dpf, gonads exhibit simple testes precursors called fetal testes, and female PGCs undergo oogonia, respectively. The testicular cords vary in size, and individual pre-spermatogonial cells are present inside the testicular cords. During this period of sex differentiation, few studies are related. After this period, the cells initiate spermatogenesis and oogenesis, which are ovulated for fertilization. Figure adapted from Yamashiro et al. (2018) [27]. Figure created in the Mind the Graph platform.

Perhaps it is possible to find germ cells around at the allantoid base until they reach the gonad [15,16]. However, there have been no reports in the literature from the first reorganization of PGC development in canines (Figure 1).

As pregnancy progress, the heartbeat can be detected using ultrasonography at 23.4 dpf [17]. Between 25–27 dpf, the cardiovascular system showed both atrial and ventricular aches, optimal globs with pigmentation, prominent forelimbs, and pelvic limbs. Interestingly, the primitive kidneys were divided into metanephros and mesonephros connected laterally with the gonadal ridge, which enlarged morphologically [13]. At this time, PGCs multiply mitotically during migration, increasing their cell population until the development of the gonads. Thus, at 30 dpf, the embryo completes the formation of the principal organs, and the PGCs undergo major morphological transformations, increasing their cell number until they reach sexual differentiation (Figure 1).

The fetal period is marked by the end of organogenesis around 35 dpf [7]. The period around 35–40 dpf is marked by the initiation of sexual differentiation, during which the cells can be morphologically distinguished as males or females. The male gonads undergo major morphological changes, medullary cords are differentiated in the seminiferous cords. The female gonadal ridge was divided into the medulla (inside the gonad) and cortex (outside of the gonad) [7,18]. The same results could be observed in humans; this separation is evident in the sixth week of pregnancy [19]. However, in mice morphogenesis development, on stage E11.5 the distinction between the cortex and the medulla is not evident [20]. Comparing canine gonadal ridge development with another domestic species, it seems that around 30–35 dpf, the morphological transformation and sex differentiation remain similar between canine and porcine species. For example, in domestic pigs, with an average gestation of 115–120 days, PGCs are found in the dorsal mesentery of the hindgut on days 18 and 20 of pregnancy and begin to arrive in the developing primordial of the genital ridge on days 23 and 24. Sexual dimorphism begins around day 30 of gestation in these species [21].

Around 45–55 dpf, gonads exhibit simple testes precursors called fetal testes, and female PGCs undergo oogenesis. The testicular cords vary in size, and individual pre-spermatogonial cells are present inside the testicular cords [7,18]. In postnatal life, the process of folliculogenesis begins when the first primordial canine follicles are observed approximately 2–3 weeks after birth [22]. Primary or early preantral follicles occur around day 120 after birth and contain small, pale oocytes [23]. According to the literature, the secondary or advanced preantral follicles contain fully grown oocytes; however, this event’s exact day or age is unknown [24]. Early antral follicles can be observed from day 120 through 160 after birth [22,23,24]. At the end, advanced antral follicles are present in bitches as young as 6 months of age during mid-proestrus [25,26] (Figure 1).

## 3. Spermatogonial Canine Stem Cells

In mammals, spermatogenesis begins in puberty. However, in fetal testis, the gonocytes (pre-spermatogonia, named as daughter cells of primordial germ) are localized to the seminiferous cord center and proliferate a few days before they enter a quiescent state. In the neonatal period of development, the gonocytes restart mitosis and generate two groups of cells, one group of cells becomes spermatogonia, and another group of cells develop to establish the SSC group [28,29]. In male canine, gonocytes differentiate in pre-spermatogonia that precede puberty to form a specific type of spermatogonia (A) [30] (Figure 1). In a Beagle dog, the number of spermatogonia and Sertoli cells increases in 2–3 months old puppies. It is possible to find single and paired spermatogonia, but aligned spermatogonia were only observed in 3 months old [31] In general, dogs reach puberty, between 6 and 24 months of life, however, this variation depends on the gender, breed, hormone levels, food availability and weight [31,32,33].

For example, in the fox terrier breed, pubertal maturation begins at five months of age, with the beginning of the phase of growth of the size testicles, and ends at nine months, when spermatogenesis is completed [34]. The puberty period is defined as the end of a maturate procedure, leading to fully-functional primary and secondary sexual organs as well as to typical sexual dimorphic behavior. Puberty in canine is characterized by the expansion of the seminiferous tubules, and germ cells move to the basement membrane, and an increase of the spermatogonia in the seminiferous tubules in the first year of life is observed [30]. During this period, physiological and hormonal changes begin, such as the production of follicle-stimulating hormone (FSH) and luteinizing hormone (LH), which exert their actions on spermatogenesis mainly through the regulation of Sertoli cell factors and regulation of sperm production [35]. According to the literature, in canine, the spermatogenesis process is initiated at 7 months of age; however, at 10–12 months of age, it is possible to verify the presence of sperm in canine ejaculate [32]. However, sexual maturity in canines occurs when spermatogenic yield reaches maximum levels (generally, 3–6 years of age). Still, there are many canine breeds and strains, and for this reason, there may be some variations in the spermatogenesis process and production of canine sperm [36].

Spermatogenesis is a physiological and cyclic process that occurs in the seminiferous tubules of adult mammals, and it can last 30–78 days [37]. In canine, the process of spermatogenesis lasts approximately 60 days, and it is the time necessary for spermatogonia to be released in the seminiferous tubules and produce sperm [36]. Therefore, spermatogenesis is maintained through the continuous activity of spermatogonial stem cells and the extraordinary interplay of autocrine, paracrine, and endocrine factors [38]. In mammals, such as canine, this process occurs in a regular system with cellular division and differentiation. The cells next to the basal membrane, called stem cell spermatogonia, gradually undergo morphological, functional, and biochemical changes [39,40]. The SSCs undergo mitosis and some cells self-renew and remain inactive, usually, these spermatogonia are stored to restore the spermatogenesis and some cells are differentiated in other types of spermatogonia will form the primary spermatocytes to continue the spermatogenesis process [40] and produce millions or billions of sperm every day (depend on each specie) [39,40].

**Figure 2:**
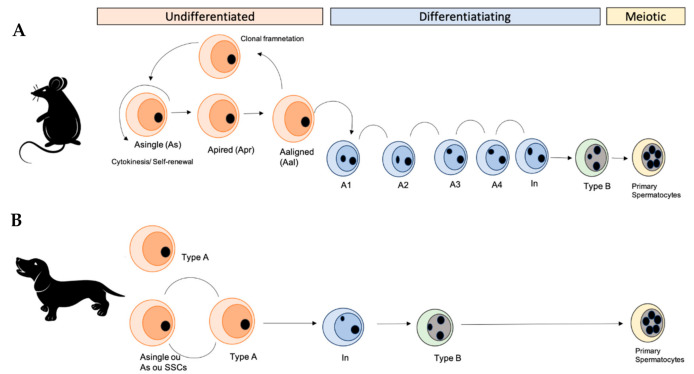
Self-renewal and differentiation of the spermatogonial population of rodents and canine. (**A**) Spermatogonial stages in rodents, showing the different subtypes of spermatogonia. Spermatogonial stem cells or Asingle spermatogonia (As) are rare cells in the testes and they can self-renew to produce two new As (self-renewing division) or for mitotic division, differentiate into spermatogonia Apaired (Apr). In addition, spermatogonia Apr remain connected by a bridge intercellularly and in the next mitotic division produce a chain of four Aaligned spermatogonia (Aal4). These As, Apr, and Aal form the population of undifferentiated spermatogonia in the seminiferous tubules. The Aal spermatogonia could undergo one or more mitotic divisions to form chains of 8, 16, and sometimes 32 cells. The last phase of this process occurs during the differentiation of spermatogonia A1. After, A1 spermatogonia undergo sequential mitotic division and produce types A2, A3, A4, Intermediate, and B spermatogonia, which divide to produce primary spermatocytes. (**B**) In canine, spermatogonia type A are stem cells (SSCs or As) that self-renew and proliferate, resulting in mitosis and differentiation into intermediate and later type B, which divide to generate primary spermatocytes (Based on the data described [36,41,46]).

In male canines, spermatogonial stages are different from the mice and other animals, such as monkeys [41]. For example, in mice, the first SSCs are found 3–8 days post-coitum (dpc) [42]. In this species, the spermatogonia divide into different subtypes of spermatogonia. Then, SSCs or Asingle spermatogonia (As) are rare cells in the testis (consisted 10% of undifferentiated spermatogonia; 1 in every 30,000 cells per testis or 0.03% of all germ cells) [40,43]. They can self-renewal and can produce two new As (self-renewing division) or by mitotic division differentiate into spermatogonia Apaired (Apr). The SSCs (As) cells can self-renew and produce two new As cells or they can by mitotic division produce a pair of spermatogonia (Apr; Apaired). Apr spermatogonia will produce two new As (self-renewing division) or remain connected by an intracytoplasmic bridge and in the next mitotic division could produce a chain of four Aligned spermatogonia (Aal4). As, Apr, and Aal form the population of undifferentiated spermatogonia in the seminiferous tubules. The Aal spermatogonia could undergo one or more mitotic divisions to form chains of 8, 16, and sometimes 32 cells. During the last phase, the differentiation into spermatogonia type A1 occurs. After, A1 spermatogonia undergo sequential mitotic division and produce types A2, A3, A4, Intermediate (In), and B spermatogonia, which divide to produce primary spermatocytes [44,45]. In the literature, it was found that in canine the first spermatogonia formed (A) will generate spermatogonia A1 which will progressively self-renew and divide into intermediary spermatogonia (type In) and type B [36]. It is known that cell type A is responsible for the formation of reserve germ cells and type B for continuing spermatogenesis [41,46] (Figure 1 and Figure 2).

However, few studies have described the types of canine spermatogonial, including spermatogonia stem cells (SSCs). Two to 7 years old beagles show eight stages of the seminiferous epithelium cycle, whereas men show 6 stages, mouse 12 stages, and rats 14 [47,48]. Each spermatogenic cycle is defined by one generation of germ cells that is a group of the cells in the same step of development, and these cells form a fixed sequence that repeats itself indefinitely (cell associations). Even, the spermatogenesis cycles are divided into a species-specific number of stages [49,50]. The stages of the spermatogenic cycle are established before puberty and may vary depending on the criteria used to characterize them [37,49,51]. In canines, the criteria were the morphology of spermatids, tubular diameter, and acrosome head [36]. However, the stages of the seminiferous epithelium cycle can undergo some variation in different species.

In the testes of adult canine the spermatogenesis is divided into 8 stages (I-VIII). In stages I and V of the seminiferous epithelium cycle, germ cells in different phases such as spermatogonia type A, intermediate, B, primary spermatocytes, and spermatids. Thus, the presence of meiotic figures of the first and second divisions as secondary spermatocytes and early-round spermatids only in stage VIII. [32,36]. The spermatogonial type A was observed in contact with the basal lamina in stage I of the cycle and after in a great number in another stage of the cycle, and in stages VII and VIII, the number substantially increased in spermatogonial type A. In the seminiferous epithelium cycle, most stages show differences in frequency relating to the breed, mostly in mongrel dogs and the American Pit Bull. However, no explanation regarding the possible mechanisms related to the acceleration of the duration of spermatogenesis in this breed was reported. The researchers suggested that the scrotum of this breed seems to be more attached to the perineal region and this anatomical peculiarity could result in an elevated scrotum temperature [36].

Research has shown that the duration of spermatogenic cycles can be changed in each breed. The spermatogenic process was separated by body weight and showed that the average cycle duration of the seminiferous epithelium for mongrel dogs, Beagle, Pinscher, Poodle, and Labrador is 13.73 ± 0.03 days [36]. In addition, the duration of each stage of the cycles showed a difference, for example in all breeds stage II is the shortest (1 day) and stage VIII is the longest (3 days). Early round and elongated spermatids, pachytene spermatocytes, a small number of intermediate spermatogonia, and type A spermatogonia were observed in stage II. However, elongated spermatids were undergoing spermiation toward the tubular lumen and Type B spermatogonia, type A spermatogonia and pre-leptotene spermatocytes (originating from Type B spermatogonia) were observed in stage IV [36]; nevertheless, 4,5 cycles are required for the whole spermatogenesis to be complete and the total length of spermatogenesis is estimated to be 61.9 days in mongrel dogs, Poodle, Pinscher, Beagle, and Labrador and approximately 30 to 78 days in others mammals [37,52].

This study noted a significant difference in the frequency of some phases of the cycle in the American Pit Bull, which reduced the total length of spermatogenesis in this breed (56.5 days) [36].

## 4. Germ Cell Signals

The beginning of PGCs development, proliferation, and differentiation is specified by signaling molecules and specific gene secretion factors. For example, in mice, the epiblast cells occur between the extraembryonic ectoderm and the visceral endoderm, forming a cylinder. Near the epiblast, the cells induce signals, which begin to emanate, and a few cells become PGCs. The other cell types are differentiated into somatic cells [53]. The specification of PGCs in mice depends on the *Fragilis* gene especially on the transcriptional factors Blimp1, Prdm14, and Tcfap2c (or AP2ɤ) [45]. Studies with knockout mice of the genes *Blimp1*, *Prdm14*, and *Tcfap2c* demonstrated an absence in the proliferation and migration of PGCs [54,55]. In addition, *NANOS3* is involved in prenatal germ cell development [56,57]. According to the literature, NANOS3 has been directly shown to function in germ cell development across diverse species from flies, worms, frogs, and mice to humans [58] (Table 1).

Another factor that assists in PGCs signaling is the bone morphogenic protein 4 later (BMP4) which is expressed in the extraembryonic ectoderm and assists in cell growth. In Mice BMP4 and BMP8B are factors that are expressed during the inducing of distal visceral endoderm formation that will establish embryo anterior–posterior axis (E5.5) besides being present in earliest cell heart precursors located on both sides of the midline in the epiblast of early gastrula stage embryos E6.5 days post-coitum (dpc) inducing appropriate mechanisms for PGCs development. Studies with mutant embryos showed a complete lack of PGCs and allantois, and the BMP8B mutants showed few or no PGCs in addition to reduced allantois size [59,60]. The low regulation of BMPs also influences SMAD proteins (SMAD 1, SMAD 5, and SMAD 8), which aid in epiblast development [61]. Consequently, abnormal expression of these proteins affects epiblast formation and PGC irregular formation [62,63] (Table 2).

In canines, the first signals or genes in PGCs have not been described in the literature. However, during migration, colonization in the gonadal ridge, and sex transformation, the interactions of PGCs can be identified with alkaline phosphatase (AP) activity and the OCT4 (or POU5F1) gene/protein [7,64]. This transcription factor, OCT4, functions as a regulatory key in the pluripotential phenotype. It is expressed in embryogenesis, more specifically in the epiblast compartment, in embryonic carcinoma cells, stem cells, and after gastrulation; its low regulation decreases, restricting its expression in germ cells [65,66,67]. It seems that the OCT4 gene in PGCs is well conserved among domestic species, such as bovine species [68,69], ovine [70], and porcine [21].

Other nuclear protein markers related to pluripotency, such as NANOG, SOX2, and STELLA, are also present during the development of PGCs. STELLA (DPPA3 or PGC7) acts on the maintenance of pluripotent cells and oocyte cells as well as the early development of PGCs [71]. In male canine PGCs, the *NANOG* and *STELLA* genes were present at 35 dpc, and the STELLA protein was detected in the late period from 45 dpc [7]. Interestingly, gene and protein profiles differed in female canine PGCs. The STELLA protein was present only in the middle period (35–40 dpf); however, there was no abundance of *the STELLA* gene in the middle and late periods (35–50 dpf) [18]. The *NANOG* gene was also present only in the middle period (35–40 dpf). This study suggests a sex difference in the presence of genes and proteins between male and female PGCs development, as observed in humans [72] (Table 3).

During the PGCs migration stage, other markers begin to be expressed. In mice, the marker anti-stage-specific embryonic antigens (SSEA1) are present in mESCs, and during PGCs migration [73]. Interestingly, SSEA1 is absent in human embryonic germ cells (hESCs), but studies on human EGCs have demonstrated positivity for this marker [74]. In canines, the SSEA1 marker is present in iPSCs and multiple cancers, such as glioblastoma, melanoma, and mammary cancer [75,76]. However, there are no data in the literature regarding SSEA1 markers in PGCs. In domestic animals, such as bovine [68,69], ovine [70], and porcine [21], PGCs can be identified by SSEA1. Another marker that plays an important role is the tyrosine kinase receptor, known as C-kit (or CD117), which is located on the cell surface. The function of the C-kit in PGCs is assisting in the colonization and development of gonadal ridges, avoiding apoptosis [77,78]. In canine PGCs, there is no date about the C-kit marker. However, in mice, C-kit is highly present during migration and colonization in the gonads, but when it initiates sexual differentiation the expression decreases [79].

Upon entering the genital ridge, the specific germ marker VASA (or DDX4) and “Deleted in Azoospermia-like” (DAZL) are conserved between vertebrate and invertebrate species [80,81,82,83]. Both markers play an essential role in PGCs development and maintenance; the absence of both transcripts is associated with infertility [84,85]. The VASA protein is present in PGCs from male and female canines. In other species, such as cattle, the first population of PGCs positive for the VASA marker was detected only after 100 days of fetal development in females, with no information during embryonic and fetal development in males [86]. As in canine and the other species, the DAZL transcript and protein have been associated as a mature PGCs marker, because the DAZL presence is related to sexual differentiation in seminiferous cords in the male embryo and primordial follicles in the female embryo [85,86,87]. The comparison with other domestic animals is limited because of the absence of literature.

In the adult period, SSCs and other types of undifferentiated spermatogonia could be positive for alpha-6 integrins (CD49f), PLZF, and GFR-α1 markers [88,89,90]. However, in domestic animals, knowledge about phenotypic spermatogonia markers is relatively limited [43,90]. For example, in canine, few studies have investigated the expression of markers in canine germ cell lines [9,32,46,91,92]. CD49f represents family integrins. In the seminiferous tubule microenvironment, inactive integrin subunits can induce cytological, chromosomal, and morphological changes in spermatogonia following detachment of SSCs from the seminiferous tubule basement membrane [93,94]. In porcine, integrin heterodimers actively function on the surface of undifferentiated SSCs [95].

In pre-pubertal canine, testes have been reported to detect proteins in spermatogonia, and specific target antigens of SSCs that were identified as VASA, ubiquitin carboxy-terminal hydrolase L1 (UCHL1), DAZL, PLZF, OCT4 (POUF5), and proto-oncogene encodes a receptor tyrosine kinase (RET) [92]. Unfortunately, they do not demonstrate SSC-specific expressions such as the glial cell-derived neurotrophic factor family receptor alpha-1 (GFRα-1), B1 and A6 integrins, and probably G-protein coupled receptor 125 (GPR 125), and RET has a sporadic detection in prepubertal canine testes. GFRα-1 and PLZF markers were found in spermatogonia of the canine prepubertal and adult testes [46]. Promyelocytic leukemia zinc finger (ZBTB16 or PLZF) is a marker of undifferentiated spermatogonia and is involved in maintaining their undifferentiated character via epigenetic regulation. GFRA1 is the co-receptor for GDNF, a Sertoli cell-derived factor that controls the balance between self-renewal and differentiation of SSCs. GDNF signaling acts via the RET tyrosine kinase present in undifferentiated type A-spermatogonia and requires a ligand-specific co-receptor GFRA1, a conserved marker to promote spermatogonial self-renewal [96]. Some authors also suggest that, apart from regulating stem cell self-renewal, GDNF also stimulates cells to progress along the differentiation pathway [97]. In some species, such as the feline, this surface marker could be used for SSCs and germ cell purification [98,99].

The VASA marker detected during the development of germ cells is conserved in mammalian. However, in mice during the adult period, the protein could be detected in testicular germ cell differentiation and was mostly expressed in spermatocytes and round spermatids in the adult testis, although it was absent in SSCs and a decrease of protein during spermatogenesis was observed. One study in canine mentioned that VASA is one of the proteins detected in SSCs [100]. Recently, VASA protein showed a positive expression in all germ cells from fetal and prepubertal testis sections in cats [101].

Research has shown that STRA8 and DAZL are essential during spermatogenesis. STRA8 is important because it encodes a protein necessary for replicating pre-meiotic DNA after being angled into the prophase. Studies in mice show that the STRA8 gene is linked to fertility, expressed in pre-meiotic cells [102,103], and it is expressed before and during spermatogonial differentiation and in spermatocytes [104,105]. DAZL is a germ cell-specific RNA-binding protein that is considered a major regulator of spermatogenesis [106]. DAZL is expressed in the nucleus of spermatogonia and is transferred to the cytoplasm of primary spermatocytes during meiosis [39]. In in vitro the canine spermatogonial cells, DAZL was detected in the membrane/cytoplasm of type A and intermediate spermatogonia from prepubertal testis, mainly in the center of seminiferous tubules in the adult testes, and DAZL was also present in differentiated spermatogonia.

In addition, C-kit (or CD117) is a member of the tyrosine kinase receptor family used in many species as a specific marker for differentiating spermatogonia and subsequent germ cell stages, such as spermatocytes and interstitial Leydig cells, but not in undifferentiated spermatogonia and Sertoli cells [107,108]. The C-kit was detected in Sertoli cells and undifferentiated spermatogonia on prepubertal, differentiated spermatogonia, and spermatocytes on pubertal of canine testes. In another study, C-kit was expressed in interstitial Leydig cells and spermatogonia in adult canine testes [109]. In mice bearing mutations, this gene causes sterility due to the developmental failure of primordial germ cells during early embryogenesis. This protein is a marker for SSC pluripotency loss, and its expression continues until meiosis is initiated [110]. The self-renewal in spermatogonia is achieved via repression of C-kit, which plays an important role in differentiation initiation [111,112].

In addition, pluripotency markers with OCT4 (POU5F1) and NANOG were responsible for maintaining the regulation of self-renewal and pluripotency and are used as stem cell markers [113,114]. In deficient mice, PGP9.5, which is resistant to the wave of germinal cell apoptosis is possibly observed during the first round of spermatogenesis. These mice have defects in sperm production, motility, and morphology [113]. In testes of Beagle dogs at 2, 3, and 12 month-old, this protein was detected only in spermatogonia located in the basal membrane of seminiferous tubules. In addition, the PGP9.5 protein was present in spermatogonia cells from testes of 4- and 5-month-old Belgian Malinois [32].

In addition, in pluripotency markers with OCT4 (POU5F1), NANOG was responsible for maintaining the regulation of self-renewal and pluripotency and is used as a stem cell marker [115]. For example, in cats, the smallest subpopulations of spermatogonial cells expressed NANOG, OCT4, and SOX2, but these genes were not specific to SSCs. Besides, in mice and humans, OCT4 is used to detected germ cells [116,117]. A few studies have described the pluripotency genes or markers in germ cells in canine, and their expression remains unclear. In canine, OCT4 protein was detected in germ cells in vitro from prepubertal and adult germ cells, and it increased over time [46]. In another study, the authors reported increased expression in fetal but not postnatal germ cells [107]. In addition, the expression of OCT4 and NANOG could vary in spermatogonia colonies in culture [118]. The pluripotent marker, NANOG, was expressed in spermatocytes (between 6 and 8 months) and adult spermatogonia and spermatids (between 1 and 5 years old) [119]. The detection of the subpopulation of human spermatogonias to OCT4 and NANOG indicates the existence of a population of cells among spermatogonia with SSC and pluripotent characteristics [117] (Table 3).

## 5. Canine Germ Cells in Vitro

**Figure 3:**
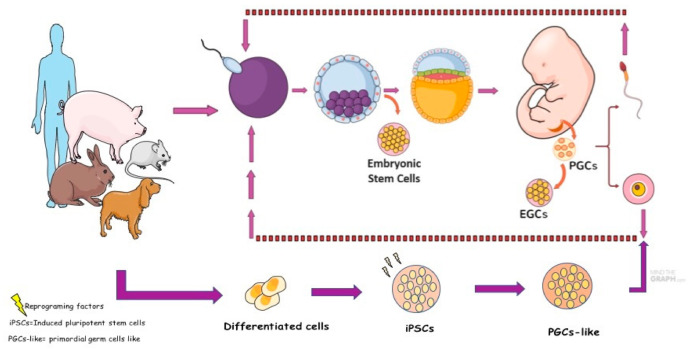
Schematic model of embryonic stem cell derivation (ESCs), primordial germ cells (PGCs), embryonic germ cells (EGCs), induced pluripotent stem cells (iPSCs), and primordial germ cells, such as PGC-like cells. The oocyte is penetrated by the sperm, resulting in zygote formation. During the initial mitotic division, the zygote develops into a 2-cell embryo, 4-cell embryo, initial morula, compact morula, and subsequent blastocyst—the internal blastocyst cell mass (ICM) turn, becomes the embryo in the future. The outer layer of the blastocyst contains some cells that are collectively called the trophoblast. Embryonic stem cells (ESCs) can be derived from a blastocyst ICM. During these cell divisions, the blastocyst expands into a bilaminar embryonic disc and is then transformed into a trilaminar embryonic disc through gastrulation. Gastrulation leads to the formation of the three somatic germ layers, ectoderm, mesoderm, and endoderm. Primordial germ cells (PGCs) arise from a population of pluripotent somatic cells in the proximal epiblast near the extraembryonic ectoderm. PGCs are multiplied by mitosis during the embryogenesis process and migrate to the embryo until they reach the gonadal ridge. Approximately between the period of migration and colonization of PGCs in the gonads, it is possible to derive embryonic germ cells (EGCs) in vitro. The other possibility of generating PGCs and gametes is by inducing a differentiated cell into iPSCs and transforming them using supplements into cells similar to PGCs or called primordial germ cells-like (PGCs-like). Figure adapted from Turnpenny (2005) [121] Figure created in the Mind the Graph platform.

PGCs in culture could increase their cellular plasticity properties and can be transformed or reprogrammed into another cell type whose name becomes: embryonic germ cell (EGCs) [120] (Figure 3). This transformation process allows the expression of pluripotential factors, the formation of embryoid bodies and teratomas in the three germinative tissues, similar to embryonic stem cells (ESCs).

In mice, the conversion of PGCs into EGCs has already been well established and characterized [122]. According to the protocols of mouse EGCs (mEGCs), the cells can survive long-term in culture, form colonies, and are positive for pluripotent markers such as ES cells, form chimeras, and possibly differentiate into gametes [123]. Canine EGCs were reported successfully; these cells presented pluripotent and germinative proteins and genes, as reported in mice, humans, and domestic animals such as pigs, rabbits, rat, goat, sheep, and buffalo [7,70,124,125,126,127,128]. Although the success reported in producing EGCs, these cells have a limited and inefficient system culture because they differentiate quickly into other cell subtypes. Furthermore, ethical accessibility in obtaining PGCs from embryos in the early stages restricts the production of EGCs. Thus, new strategies for obtaining germ cells were developed from ESCs, induced pluripotent stem cells (iPSCs) cells, and intermediate embryonic stem cell type (XPSCs).

Studies have shown that induced pluripotent stem cells (iPSCs) and ESCs from both mice and humans can be reprogrammed into a PGCs population. The first induction of PGC-like cells was reported in mice, which converted iPSCs and ESCs into the status of epiblastic cells such as EpiLCs and subsequently induced PGC-like cells [129]. Nevertheless, human iPSCs and ESCs can be induced into PGC-like cells [130,131]. Human PGC-like proteins showed different key factors that regulate germ cell induction, diverting from mouse PGC-like cells. Recently, XPSCs from mice, horses, and humans showed that they could contribute to chimeras and create precursors to sperm and eggs in a culture dish [132]. Based on this principle, it is important to study different species of animals to understand the basic principles of germ cell formation and transition in mammals. There have been no reports of canine ESC, iPSCs, and XPSCs induced into PGC-like cells, possibly because of the difficulty in establishing and maintaining canine ESCs and iPSCs in culture, as reported in the previous studies [133]. However, two pioneering studies differentiated canine adipose mesenchymal stem cells (ADMSCs) into PGC-like cells. For the first time, canine ADMSCs stimulated with bone morphogenic protein (BMP4) can generate PGC-like cells, and the cells showed the presence of PRDM1, PRM14, DMRT1, and PLZF markers [8]. The same research group published two other studies showing that overexpression of CD61 can promote human umbilical cord mesenchymal stem cells (hUC-MSCs) and canine ADMSCs into PGC-like cells [134]. The results showed that canine PGC-like cells had the presence of *PRDM1*, *PRDM14*, *AP2-γ*, *CD49F*, *SOX2*, and *NANOG* genes. In addition, immunofluorescence assays showed the classical germ cell markers DAZL and VASA. In summary, studies on canine germ lineage from ESCs and iPSCs could be an interesting source in basic veterinary research because of the lack of information at the beginning of the development of PGCs in vivo. In addition, since canines have similar genetics and diseases as humans, research with canine PGC-like cells could be a new animal model and one opportunity to generate in vitro gametes.

Research on SSCs has been increasing because of the easy access to cells and culture in vitro. In addition, the study of SSCs allows genetic manipulation to enhance desired traits or understand the biological and endocrinological features of canine and other species. Therefore, many attempts have been made to establish SSC cultures in domestic animals, such as the utilization of specific growth factors, serum, and conditioned medium originating from testicles [135,136,137,138].

Isolation and culture of canine SSCs were reported in one variety of canine breeds in the age range of 3–5 months [92]. In early cultures, most of the cells adhered to the substratum but maintained a relatively rounded morphology compared with elongated fibroblasts. The rounded cells were often arrayed as connected chains, reminiscent of the syncytial chains of type A-aligned spermatogonia (SPG) in vivo. Additionally, spermatogonial cell cultures from Beagle canine 2-, 3-, and 12 months old were isolated and cultivated with a combination of supplements, such as mouse epidermal growth factor (mEGF), basic fibroblast growth factor (bFGF), glial cell-derived neurotrophic growth factor (GDNF), and leukemia inhibitory factor (LIF). The results showed that the DMEM medium with 5–10% of fetal bovine serum supplemented with GDNF and FGF, or LIF and EGF, can maintain the SSCs in vitro for a long period [92].

Recently, the protocol for the isolation, culture, and characterization of canine SSCs from mongrel dogs showed that the cells were not adherent to the plate, with a round shape, and after 5 days of cultivation, the cells formed clusters such as a colony under the monolayer of fibroblast cells. The SSCs were capable of self-renewal and maintenance in vitro to present phenotypic characteristics of GFRA1 and PLZF in culture. In addition to the demonstration, the combination of the two techniques of enrichment, differential plating, and the percoll density gradient, showed the efficiency of purification and isolation of canine SSCs [46].

However, few studies have consolidated canine SSC cultures. Nonetheless, some researchers have discovered important results that supported the SSCs culture for the long term. The progression of SSCs in vitro culture research has a valuable precedent for reproductive science on cellular therapy, cell transplantation, testis tissue xenografting, and testis cell aggregate implantation. Therefore, new investigations should be performed once it is known that the niche in vitro, where these cells are maintained influences in vivo behavior.

## 6. Conclusions

Germ cells are among the most important cells that can transmit genetic and epigenetic information to the next generation. Studies involving germ cells improve the knowledge in biotechnology of the reproduction and production of PGCs in vitro. In vitro generation of PGCs-like and function sperms from SSCs could be transported in reproductive science to use in vitro fertilization. Besides, in vitro functional gametes could assist in the conservation of endangered species in the future. However, few studies have consolidated on domestic animals and canine species. Then cooperative studies perhaps enable the advance of germ cell specification and signals between canine and other mammals.

## Figures and Tables

**Table 1:** A summary of relevant genes for the specification of human PGCs.

Classification	Genes	3–5 Weeks	5–8 Weeks	9–10 Weeks (Female)	9–10 Weeks (Males)
PLURIPOTENT	*POU5F1*	+	+	+	+
	*NANOG*	+	+	+	+
	*PRMD14*	+	−	−	−
GERM CELLS (INITIAL)	*BLIMP1*	+	−	−	−
	*CD38*	−	+	−	−
	*NANOS3*	−	+	−	−
GERM CELLS (LATES)	*DDX4*	−	+	+	+
	*DAZL*	−	−	−	+
	*SCP3*	−	−	+	−
ENDODERMIC	*SOX17*	+	−	−	−
MESODERMIC	*T*	+	−	−	−
	*CKIT*	−	+	+	−

**Table 2:** A summary of relevant genes for the specification of mouse PGCs.

Classification	Genes	E 3.5	E 5–6.0	E 7.25–7.5	E 10.5	E 14.5 Female	E 14.5 Male
PLURIPOTENT	*POU5F1*	+	+	+	+	−	+
	*NANOG*	+	−	+	+	+	+
	*SOX2*	+	+	+	+	+	+
GERM CELLS (INITIAL)	*BLIMP1*	−	−	+	+	−	−
	*FRAGILIS*	+	+	+	+	+	?
	*NANOS3*	--	−	+	+	?	?
GERM CELLS (LATES)	*DDX4*	−	+	+	+	+/−	
	*DAZL*	−	−	−	−	+	+
	*SCP3*	−	−	−	−	+	+
MESODERMIC	*T*	−	+/−	+	−	−	−

**Table 3:** A summary of relevant genes for the specification of domestic animals PGCs.

Classification	Genes	Bovine	Ovine	Porcine	Rabbit	Canine
PLURIPOTENT	*POU5F1*	+	+	+	?	+
	*NANOG*	?	+	+	?	+
	*SOX2*	?	+	?	?	?
	*STELLA*	?	?	?	?	+
GERM CELLS	*PG 2*	?	?	?	+	?
	*CKIT*	+	+	?	?	?
	*SSEA1*	+	+	+	?	?
	*SSEA4*	?	+	?	?	?
	*EMA1*	?	?	+	?	?
	*DDX4*	?	?	+	?	+
	*DAZL*	?	?	?	?	+
